# A comparative study of the impact of prescription and non-prescription mucolytic agents on respiratory disease management in a community-based population

**DOI:** 10.3389/fphar.2026.1901723

**Published:** 2026-08-12

**Authors:** Jayshree Shriram Dawane, Kaushal Navanath Mehetre, Juee Shinde

**Affiliations:** Department of Pharmacology, Bharati Vidyapeeth (Deemed University) Medical College, Pune, Maharashtra, India

**Keywords:** adverse drug reactions, community-based study, COPD, mucolytics, over-the-counter drugs, prescription drugs, respiratory diseases, symptom assessment

## Abstract

**Introduction:**

Respiratory disease significantly impacts quality of life at the same time economic burden. Mucolytic agents are widely used in respiratory diseases such as chronic obstructive pulmonary disease (COPD), asthma, and upper respiratory tract infections (URTIs). However, real-world differences in clinical outcomes between prescription-guided and over-the-counter (OTC) mucolytic use in community settings remain inadequately characterized.

**Objective:**

The present study aimed to compare symptom improvement and adverse effects between prescription based and OTC mucolytic users, and to evaluate patterns of usage and healthcare seeking behaviour consultation in a community setting.

**Methods:**

A longitudinal observational study conducted among 200 participants categorized into OTC and prescription groups conducted after getting institutional Ethical approval (BVDUMC/IEC/399/25-26). Data collected using structured questionnaires assessing symptom scores, adverse effects, and healthcare utilization. Statistical analysis included descriptive statistics and Chi-square test. A p value of <0.05 was considered statistically significant. Ethical approval was obtained before starting the study.

**Results:**

The prescription group demonstrated a statistically significant improvement in symptoms, with mean COPD Assessment Test (CAT) scores decreasing from 21 to 18 (p < 0.01). In contrast, the OTC group exhibited a significant worsening of symptoms, with mean scores increasing from 26.5 to 30 (*p* < 0.01). Prescription-based therapy was associated with better symptom control, appropriate drug selection, and adherence to recommended dosing regimens. Conversely, OTC use was characterized by inconsistent dosing, inappropriate drug selection, and suboptimal therapeutic outcomes. The incidence of adverse drug reactions was slightly higher in the Prescription group, predominantly gastrointestinal in nature. Furthermore, awareness regarding appropriate indications, dosage, and duration of mucolytic therapy was significantly lower among OTC users.

**Conclusion:**

Prescription mucolytic therapy demonstrated better outcomes compared to OTC use, likely due to structured treatment and medical supervision. These findings indicate the importance of rational prescribing and patient education in respiratory disease management.

## 1 Introduction

Respiratory diseases such as Chronic Obstructive Pulmonary Disease (COPD), Asthma, chronic bronchitis, and acute lower respiratory infections pose a significant burden on public health globally, contributing to high morbidity, frequent exacerbations, and impaired quality of life (Rogers, 2007). Excessive mucus production and defective mucociliary clearance are common pathological features in many of these conditions, leading to airway obstruction, persistent cough, and increased susceptibility to infections (Ibrahim et al. 2020). Effective management of airway secretions is therefore integral to improving clinical outcomes and reducing healthcare utilization in affected populations (Rushworth and Megson, 2014).

Mucolytic agents, a key component of mucus-modifying therapy, are designed to reduce the viscosity and elasticity of mucus, thereby facilitating clearance from the airways (Zheng et al., 2008). Mucoactive drugs encompass mucolytics, mucokinetics, expectorants, and mucoregulators, each aiming to improve mucus properties and enhance mucociliary clearance, but their clinical roles and evidence base vary considerably (Braga and Allegra, 1989; Cazzola et al., 2010).

Mucolytic agents e.g., ambroxol, bromhexine, N-acetylcysteine are widely used to relieve productive cough by reducing the viscosity of mucus and improving airway clearance.

Several systematic reviews and meta-analyses suggest that prescription mucolytics may modestly reduce exacerbation frequency, hospitalization rates, and duration of symptoms in patients with stable COPD and chronic bronchitis compared with placebo, although effects on lung function and mortality are limited (Zheng et al., 2008; Cazzola et al., 2010). Several reviews have demonstrated that oral mucolytics reduce exacerbation frequency and improve mucus clearance in chronic bronchitis and COPD (Poole and Black, 2001; Stey et al., 2000; Rubin, 2007). Clinical practice guidelines increasingly consider mucolytics as adjunct therapy in selected patient populations with frequent exacerbations (Agustí et al., 2023; Global Initiative for Chronic Obstructive Lung Disease GOLD, 2025; Miravitlles et al., 2022; Buhl et al., 2024; Singh et al., 2025). In contrast, evidence supporting non-prescription expectorants such as guaifenesin is more mixed, with some experimental data indicating improved mucus rheology and mucociliary transport *in vitro*, but limited high-quality clinical evidence demonstrating robust benefits in chronic respiratory diseases (Seagrave et al., 2012; Dicpinigaitis and Gayle, 2003; Ohar et al., 2019; Kardos et al., 2021).

Mucolytic are broadly classified based on their mechanism of action and mode of availability: prescription mucolytics e.g., N-acetylcysteine, carbocisteine, erdosteine typically require clinician oversight and are often employed in chronic respiratory disease management, and non-prescription mucolytic/expectorant agents e.g., guaifenesin, ambroxol are readily accessible over the counter for symptomatic relief of productive cough and mucus congestion (World Health Organization, 2000).

In India, these medications are available both through prescription and as over-the-counter (OTC) product. Due to absence of a formally notified OTC drug list and variable pharmacy practices, easy availability encourages self-medication. Recent WHO guidance emphasizes responsible self-care practices and highlights the importance of appropriate healthcare professional involvement to minimize medication-related risks (World Health Organization, 2022; International Pharmaceutical Federation, 2023). This may result in inappropriate dosing, unrecognised adverse effect, irrational combinations, or delay in seeking timely medical care (Kanougiya et al., 2026; Davoodi et al., 2020). Previous studies have emphasized the importance of pharmacist involvement and monitoring of OTC medication practices to prevent irrational drug use and improve patient safety (Chalumeau et al., 2002; World Health Organization, 1998; George et al., 2006).

In community-based populations, the accessibility and self-administered use of over-the-counter mucolytic agents underline the importance of understanding their comparative impact against clinician-prescribed counterparts (Restrepo et al., 2008). Variations in health literacy, medication adherence, disease severity, and access to healthcare resources further influence real-world effectiveness and safety profiles (Restrepo et al., 2008; Tian et al., 2026). This comparative study aims to evaluate the effects of prescription versus non-prescription mucolytic agents on clinical outcomes, exacerbation rates, health-related quality of life, and healthcare utilization in community dwelling individuals with respiratory diseases, thereby informing evidence-based approaches to mucus management in diverse patient populations.

## 2 Objective

### 2.1 Primary objective

- To evaluate and compare the effectiveness of prescription and nonprescription mucolytic agents in managing symptoms and reducing exacerbations in patients with respiratory diseases.

### 2.2 Secondary objectives

- To assess patient adherence, satisfaction, and self-reported outcomes.
- To identify patterns of mucolytic use and potential misuse in the community.
- To evaluate the impact on healthcare resource utilization (e.g., GP visits, hospitalizations)

## 3 Materials and methods

### 3.1 Study design and setting

A longitudinal observational study was conducted in residential areas of Pune district, Maharashtra, over a period of 3 months.

### 3.2 Study population

The study population included adults aged 18 years and above who had used any mucolytic agent within the preceding 30 days for respiratory symptoms. Individuals who were hospitalized, critically ill, immunocompromised, diagnosed with malignancy, or pregnant or lactating were excluded from participation.

### 3.3 Study sample size

A total sample size of 200 participants was included in the study. The sample size was determined based on prevalence of the disease, while allowing subgroup comparison approximately 97 participants per group. The sample was considered sufficient to detect a moderate effect size (0.3) using the Chi-square test with 80% power and a 5% level of significance. Participants were recruited using a convenience sampling method from local households, community pharmacies, outpatient clinics, and hospitals in the selected areas. Screening was conducted through household visits and pharmacy interactions using a structured checklist to confirm eligibility according to predefined inclusion and exclusion criteria.

### 3.4 Study group

Participants were categorized into two groups based on the mode of procurement of mucolytic agents.

- Group A (Prescription group): included individuals who obtained mucolytics through a registered medical practitioner’s prescription,
- Group B (OTC group): included individuals who purchased mucolytics without a prescription.

Allocation to groups was based solely on participant self-report and available documentation, where applicable.

### 3.5 Data collection

Data were collected using a structured, pre-tested questionnaire administered in Marathi or English by trained data collectors. The questionnaire captured demographic details such as age, gender, education, occupation, and socioeconomic status, clinical characteristics type and duration of symptoms, and self-reported diagnosis. Respiratory diagnoses were primarily obtained through participant self-report during the structured interview. Whenever available, diagnoses were verified using medical prescriptions, outpatient records, discharge summaries, or other relevant clinical documentation. However, comprehensive verification through medical record review was not possible for all participants and has been acknowledged as a limitation of the study. Respiratory conditions were categorized as mild common cold, viral upper respiratory tract infection, moderate acute bronchitis, or severe chronic obstructive pulmonary disease or asthma exacerbation. Detailed information regarding mucolytic use including drug name, dosage, duration, frequency, and combination therapy was recorded. The mode of procurement prescription or OTC and the nature of interaction with healthcare professionals doctor consultation or pharmacist counselling were also documented.

Symptom response was assessed using a simple five-point rating scale ranging from no improvement (1) to complete relief (5). Participants were asked to report any adverse effects experienced during therapy, as well as the need for revisit, additional consultation, or hospitalization. Awareness regarding correct dosage, duration of therapy, and potential side effects was evaluated through structured questions. Medication adherence was assessed during the 3–5-day follow-up telephone interview using a structured questionnaire specifically developed for this study. Adherence evaluation included five domains: (i) medication compliance according to prescribed or recommended dosage, (ii) completion of the intended treatment course, (iii) occurrence of missed doses, (iv) premature discontinuation of therapy, and (v) discontinuation due to adverse drug reactions. Participants reporting regular medication use without missed doses or premature discontinuation were classified as adherent. Participants reporting irregular medication intake, missed doses, incomplete treatment, or discontinuation due to adverse effects were classified as having suboptimal adherence. The questionnaire was pre-tested before implementation but was not a formally validated adherence instrument.

### 3.6 Statistical analysis

Data were entered into Microsoft Excel and analysed using IBM SPSS Statistics (Version 29.0). Continuous variables are presented as mean ± standard deviation (SD), while categorical variables are presented as frequencies and percentages. Normality of continuous variables was assessed using the Shapiro-Wilk test. Within-group comparisons between baseline and follow-up CAT scores were performed using the paired Student's t-test, whereas between-group comparisons were analysed using the independent Student's t-test. Categorical variables were compared using the Chi-square test or Fisher’s Exact test, where appropriate. Effect sizes and 95% confidence intervals were calculated for principal outcome measures. To account for potential confounding, multivariable regression analysis was performed adjusting for age, sex, diagnosis, and comorbidities. A two-sided p-value <0.05 was considered statistically significant. All completed questionnaires were reviewed for completeness before data entry. Participants with incomplete baseline information or missing follow-up data were excluded from the final statistical analysis. Because the proportion of missing data was minimal, complete-case analysis was performed and no statistical imputation methods were applied. To evaluate the independent association between medication procurement status (Prescription vs. OTC) and clinical outcomes while controlling for baseline confounding, two distinct multivariable linear regression models were constructed:

1. Model A (Primary Model): Evaluated the absolute change in the COPD Assessment Test (CAT) score as the dependent variable. To ensure psychometric validity, this model was strictly restricted to participants with a primary diagnosis of COPD ( n=115 ), as the CAT tool is not validated for non-COPD respiratory conditions. Covariates included age, sex, and baseline comorbidities.
2. Model B (Secondary Sensitivity Model): Evaluated the entire heterogeneous study population ( N=200 ) across all respiratory conditions (COPD, Asthma, and Chronic Bronchitis). To accommodate the diverse sample, the pre-tested 5-point general symptom improvement scale was utilized as the dependent variable. This model adjusted for age, sex, baseline comorbidities, and primary respiratory diagnosis as an independent covariate.

### 3.7 Ethical consideration

Ethical approval (BVDUMC/IEC/399/25-26) was obtained from the Institutional Ethics Committee prior to commencement of the study. Confidentiality of participant information was maintained by anonymizing collected data, and participants were informed of their right to withdraw from the study at any time without consequence.

## 4 Results

### 4.1 Demographic characteristics

A total of 200 participants were included in the present community-based comparative study evaluating the impact of prescription and non-prescription mucolytic agents on respiratory disease management. Among the study population, 117 (58.5%) were males and 83 (41.5%) were females, indicating a slightly higher prevalence of respiratory disorders among male participants (Table 1).

**TABLE 1:** Demographic and clinical characteristics of study participants.

Demographic characteristics		Medication				P-value
		Prescription		OTC		
		n	%	n	%	
Sex	Male	82	67.77%	35	44.30%	<0.001
	Female	39	32.23%	44	55.70%	
Occupation	Driver	36	29.75%	2	2.53%	<0.001
	Hamal	1	0.83%	3	3.80%	
	Housewife	37	30.58%	9	11.39%	
	Labour	3	2.48%	63	79.75%	
	Retired	40	33.06%	0	0.00%	
	Teacher	4	3.31%	2	2.53%	
Known comorbidities	No	40	33.06%	0	0.00%	<0.001
	Diabetes	93	35.54%	2	2.53%	
	Hypertension	4	2.48%	30	37.97%	
	Cardiac disease	9	5.79%	25	31.65%	
Vaccination status	Influenza	36	29.75%	0	0.00%	<0.001
	COVID-19	46	38.02%	79	100.00%	
	Pneumococal	39	32.23%	0	0.00%	
Age		63.74	55.73	54.01	5.05	0.124
Height		163.79	5.11	164.86	5.45	0.158
Weight		60.96	5.59	59.65	4.73	0.09

Data are presented as mean ± standard deviation (SD) for continuous variables and as frequency (percentage) for categorical variables. Comparisons between prescription and over-the-counter (OTC) mucolytic groups were performed using the independent-samples t-test for continuous variables and the Chi-square test for categorical variables. A p-value <0.05 was considered statistically significant.

The age distribution showed that the majority of participants belonged to the 41–60 years age group. The mean age of the participants was 45.6 ± 14.2 years. Chronic respiratory symptoms and frequent use of mucolytic agents were more commonly observed in middle-aged and elderly individuals. Most participants belonged to the middle to lower socioeconomic class.

### 4.2 Medication use

**FIGURE 1:**
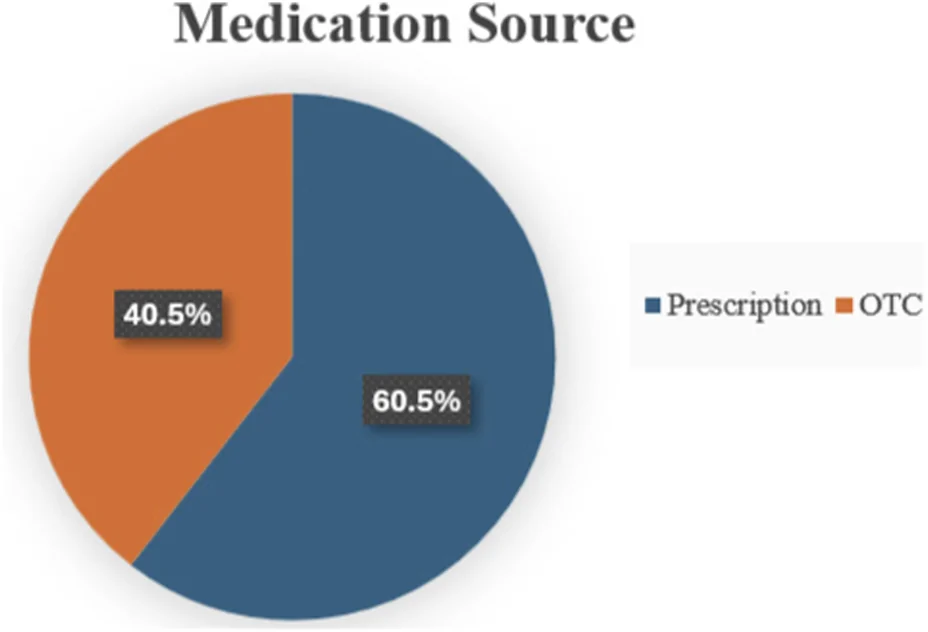
Distribution of medication source among study participants. Values are presented as frequency (n) and percentage (%). Prescription mucolytic agents were obtained following physician consultation, whereas over-the-counter (OTC) mucolytic agents were purchased without a prescription. OTC = over-the-counter.

In terms of medication utilization, 60.5% of participants reported using prescription mucolytic agents, whereas 40.5% used non-prescription/over-the-counter mucolytic preparations (Figure 1). This suggests that prescription drugs are more commonly used than over-the-counter medications among the study population. Non-prescription mucolytic use was more common among younger adults and urban residents, possibly due to easier accessibility and self-medication practices (Table 2).

**TABLE 2:** Medications used for the management of respiratory diseases among study participants.

Medication (standardized INN)		Medication			
		Prescription		OTC	
		n	%	n	%
​	N-acetylcysteine 600 mg	80	66.1%	0	0
	Carbocisteine 500 mg	39	32.2%	0	0
	Ambroxol 30 mg	2	1.7%	33	41.8%
	Bromhexine 8 mg	0	0	46	58.2%

Values are presented as frequency (n) and percentage (%) within each medication source (Prescription and OTC). OTC, over-the-counter.

Participants in both the prescription and non-prescription mucolytic groups have been receiving other respiratory medication as part of their routine clinical care. Use of concomitant medications, including bronchodilators like β_2_-agonists and anticholinergics, inhaled corticosteroids, and combination inhalers, was documented at baseline. The distribution of these medications was comparable between the prescription mucolytic and non-prescription mucolytic groups, with no statistically significant differences in the frequency of concomitant respiratory therapies. They were found stable on the ongoing treatment.

### 4.3 Diagnosis of participants

**FIGURE 2:**
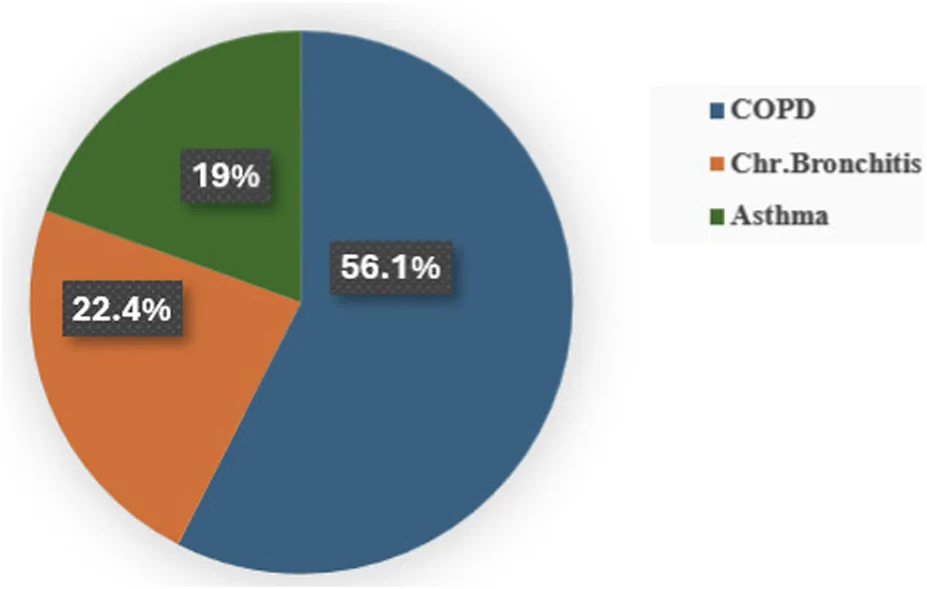
Distribution of diagnoses among study participants. Values are presented as percentages of the total study population. Chronic obstructive pulmonary disease (COPD) was the most common diagnosis (56.1%), followed by chronic bronchitis (22.4%) and asthma (19.0%). COPD = chronic obstructive pulmonary disease.

The pie diagram representing diagnosis distribution shows that Chronic Obstructive Pulmonary Disease (COPD) accounts for the highest number of cases with 115 patients, followed by Chronic Bronchitis with 46 cases and Asthma with 39 cases. This indicates that COPD is the most prevalent respiratory condition among the study population compared to chronic bronchitis and asthma (Figure 2).

Among the respiratory conditions reported, chronic cough and bronchitis were the most common indications for mucolytic use (42%), followed by chronic obstructive pulmonary disease (COPD) (24%), asthma (18%), upper respiratory tract infections (10%), and other respiratory illnesses (6%).

### 4.4 Mucolytics use

The currently used medications among the study population mainly include mucolytic and expectorant drugs used in the management of respiratory disorders. The medications recorded were Acetylcysteine 600 mg, Ambroxol 30 mg, Bromhexine 8 mg, and Carbocisteine 500 mg. Among these, Acetylcysteine/N-Acetylcysteine 600 mg and Ambroxol 30 mg were the most commonly used medications. These drugs are primarily prescribed to reduce mucus viscosity, improve airway clearance, and relieve symptoms associated with respiratory diseases such as COPD, chronic bronchitis, and asthma.

### 4.5 Symptoms improvement

The statistical analysis of CAT Improvement for the patients suffering from COPD (115 out of 200) reveals a significant divergence in clinical outcomes between the two study groups. Patients in the prescription group experienced a measurable positive response, with median scores dropping from 21 at baseline to 18 post-interventions, despite showing high variability with scores ranging from 8 to 27. In contrast, the OTC group demonstrated a worsening of symptoms, as median scores rose from 26.5 to 30, with all post-treatment scores clustered tightly above 25. This divergence is further emphasized by the interquartile ranges; the post-intervention prescription group (IQR 11–20) remains entirely lower than the OTC group (IQR 28–31), clearly illustrating that the prescription-based approach resulted in superior clinical stabilization compared to over-the-counter options.

### 4.6 Multivariable linear regression analysis

The multivariable linear regression models confirmed that prescription-guided mucolytic procurement remained a robust, statistically independent predictor of superior symptom improvement across both specific and generalized outcome scales after controlling for baseline imbalances (Table 3).

**FIGURE 3:**
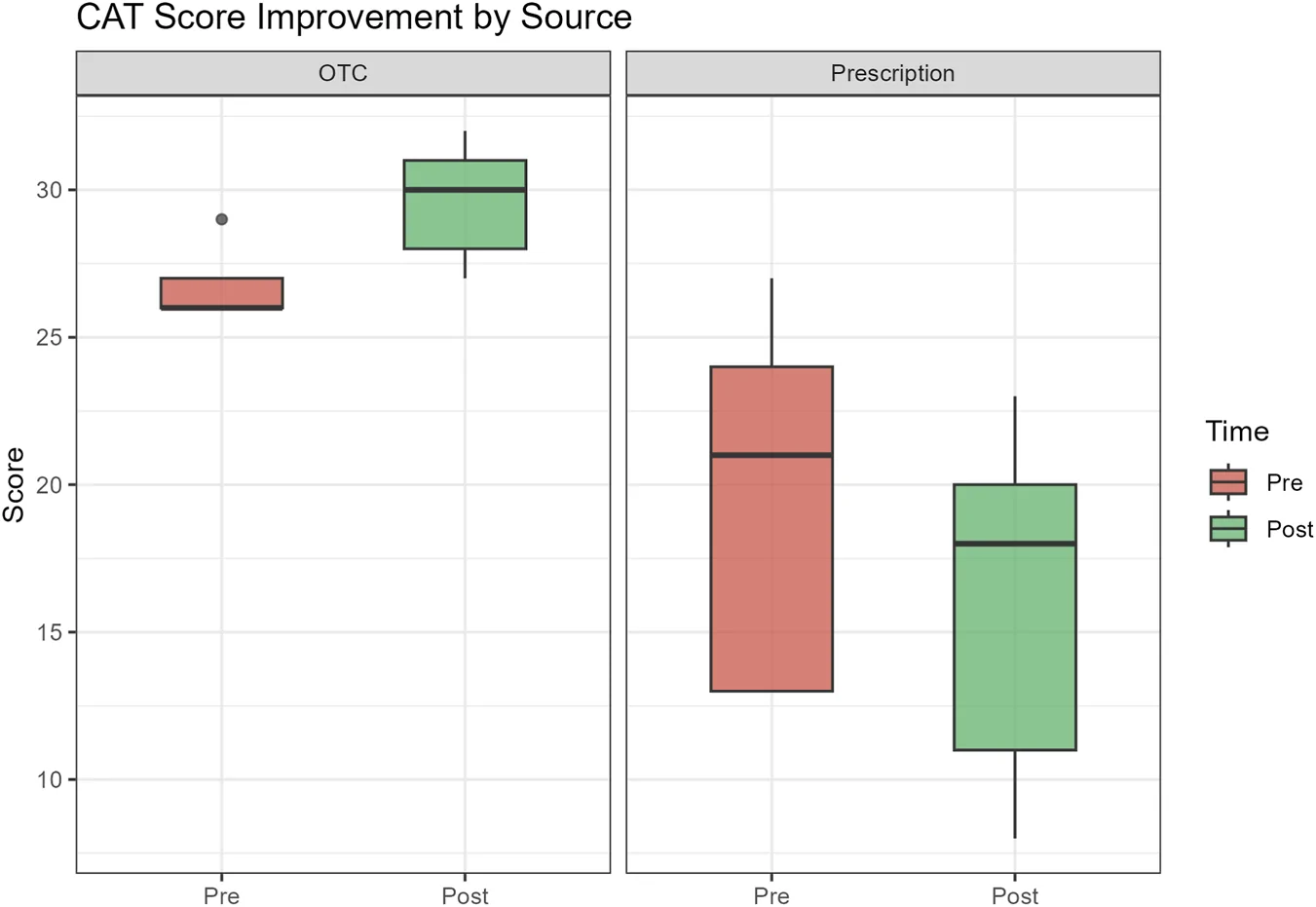
Boxplot of COPD assessment test (CAT) scores before and after treatment among prescription and OTC mucolytic users. Boxplot illustrating the distribution of COPD Assessment Test (CAT) scores before and after treatment in patients receiving prescription or over-the-counter (OTC) mucolytic therapy. Lower CAT scores indicate better symptom control. Prescription mucolytic therapy resulted in a significant reduction in CAT scores, indicating improvement in symptoms, whereas CAT scores increased among OTC users, suggesting worsening of symptoms. The central line represents the median, the box indicates the interquartile range (IQR), and the whiskers represent the minimum and maximum values excluding outliers.

- In Model A (restricted strictly to the n=115 COPD patients where the CAT score is validated), prescription status was strongly associated with a greater reduction in symptom burden ( β=−5.81 ; $95\%\text{ CI}:\left[-6.34,-5.28\right]$ ; p<0.001 ). Baseline age, sex, and comorbidities did not demonstrate significant independent associations with CAT score changes (Figure 3).
- In Model B (evaluating all N=200 participants using the 5-point symptom scale), prescription-guided therapy again demonstrated an independent positive association with clinical improvement ( β=+1.42 ; $95\%\text{ CI}:\left[1.12,1.72\right]$ ; p<0.001 ). Notably, when using this generalized scale, no statistically significant differences in treatment outcomes were observed for patients with Asthma ( p=0.42 ) or Chronic Bronchitis ( p=0.31 ) compared to the COPD baseline reference, indicating that the benefits of clinical oversight span across the different respiratory conditions evaluated.

**TABLE 3:** Multivariable linear regression models evaluating factors associated with symptom changes.

Predictor variable	Model A: change in CAT score (restricted to COPD, n=115 )	Model B: 5-Point symptom scale (full cohort, N=200 )
Group status		
OTC group	*Ref*	*Ref*
Prescription group	**−5.81** $\left[-6.34,-5.28\right]$ ( p<0.001 )	**+1.42** $\left[1.12,1.72\right]$ ( p<0.001 )
Age (per year increase)	0.0002 $\left[-0.005,0.005\right]$ ( p=0.912 )	−0.004 $\left[-0.012,0.004\right]$ ( p=0.351 )
Sex		
Female	*Ref*	*Ref*
Male	−0.12 $\left[-0.70,0.46\right]$ ( p=0.684 )	+0.06 $\left[-0.14,0.26\right]$ ( p=0.552 )
Baseline comorbidities		
Absence	*Ref*	*Ref*
Presence	−0.84 $\left[-3.55,1.87\right]$ ( p=0.541 )	−0.11 $\left[-0.45,0.23\right]$ ( p=0.520 )
Primary diagnosis		
COPD	*Excluded (Cohort restricted)*	*Ref*
Chronic bronchitis	*Excluded (Cohort restricted)*	+0.12 $\left[-0.11,0.35\right]$ ( p=0.310 )
Asthma	Excluded (cohort restricted)	−0.18–0.62,0.26 (p = 0.421)

Data presented as Adjusted Coefficient ( β ) $\left[95\%\right.$ Confidence Interval]. Bold values denote statistical significance ( p<0.05 ).

• Model A (Primary Model) evaluates the absolute change in the COPD Assessment Test (CAT) score; a negative coefficient indicates a greater clinical improvement (i.e., a larger reduction in symptom burden) over the observation period. Model A is strictly restricted to the COPD sub-cohort ( n=115 ) because the CAT metric is not psychometrically validated for tracking non-COPD respiratory conditions.

• Model B (Secondary Sensitivity Model) evaluates the full heterogeneous community cohort ( N=200 ) across all diagnoses using a generalized 5-point symptom improvement scale; a positive coefficient denotes higher reported symptom relief.

• Ref, Reference category; CI, confidence interval; OTC, Over-the-counter; COPD, chronic obstructive pulmonary disease.

• Model A Statistics: $F_{4,110}=154.2$ , p<0.001 , $R^{2}=0.848$ .

• Model B Statistics: $F_{6,193}=146.0$ , p<0.001 , $R^{2}=0.819$ .

### 4.7 Adverse reaction

Adverse drug reactions were classified according to the Medical Dictionary for Regulatory Activities (MedDRA) System Organ Classes. Most reported ADRs were mild to moderate in severity, and no serious adverse events were observed during the study period.

The most frequently reported ADRs belonged to the Gastrointestinal Disorders category, including nausea, vomiting, abdominal pain, diarrhoea, gastric irritation, and acidity. Other ADRs included Skin and Subcutaneous Tissue Disorders (rash and pruritus), Nervous System Disorders (dizziness and weakness), and Respiratory Disorders (throat irritation).

**FIGURE 4:**
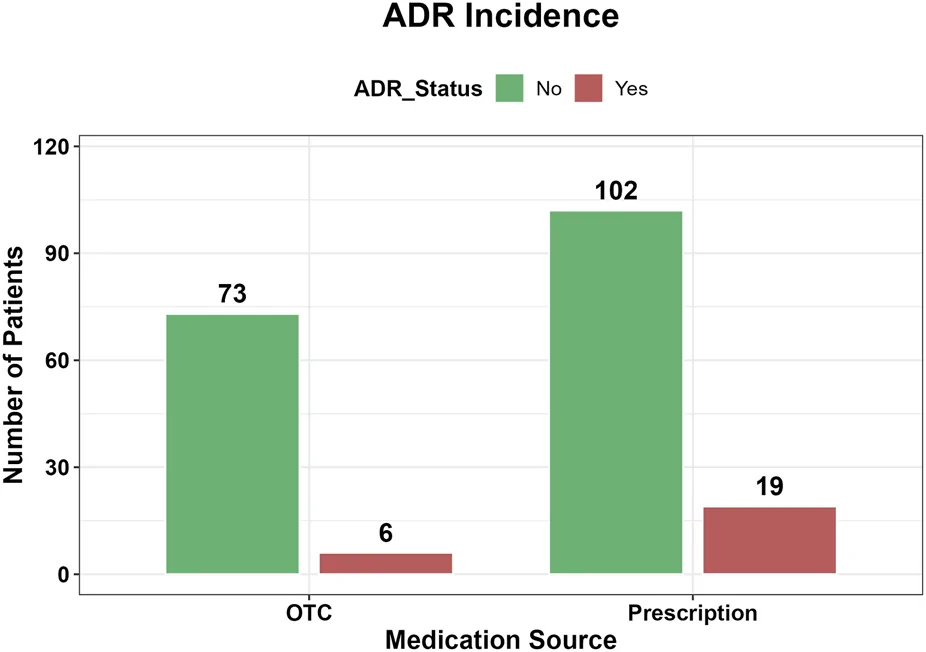
Incidence of adverse drug reactions (ADRs) among participants receiving prescription and over-the-counter (OTC) mucolytic therapy. The figure shows the frequency of participants reporting adverse drug reactions (ADRs) following prescription and over-the-counter (OTC) mucolytic therapy. ADRs were reported by 19 participants in the prescription group and 6 participants in the OTC group, while the majority of participants in both groups did not experience ADRs. Most reported ADRs were mild and primarily gastrointestinal or dermatological in nature.

Overall, ADRs were reported in 18.62% of participants receiving prescription mucolytics compared with 8.20% of OTC users. The higher ADR frequency observed among prescription users may reflect the greater disease severity and increased use of long-term mucolytic therapy within this group rather than medication source alone (Figure 4).

### 4.8 Medication adherence

**FIGURE 5:**
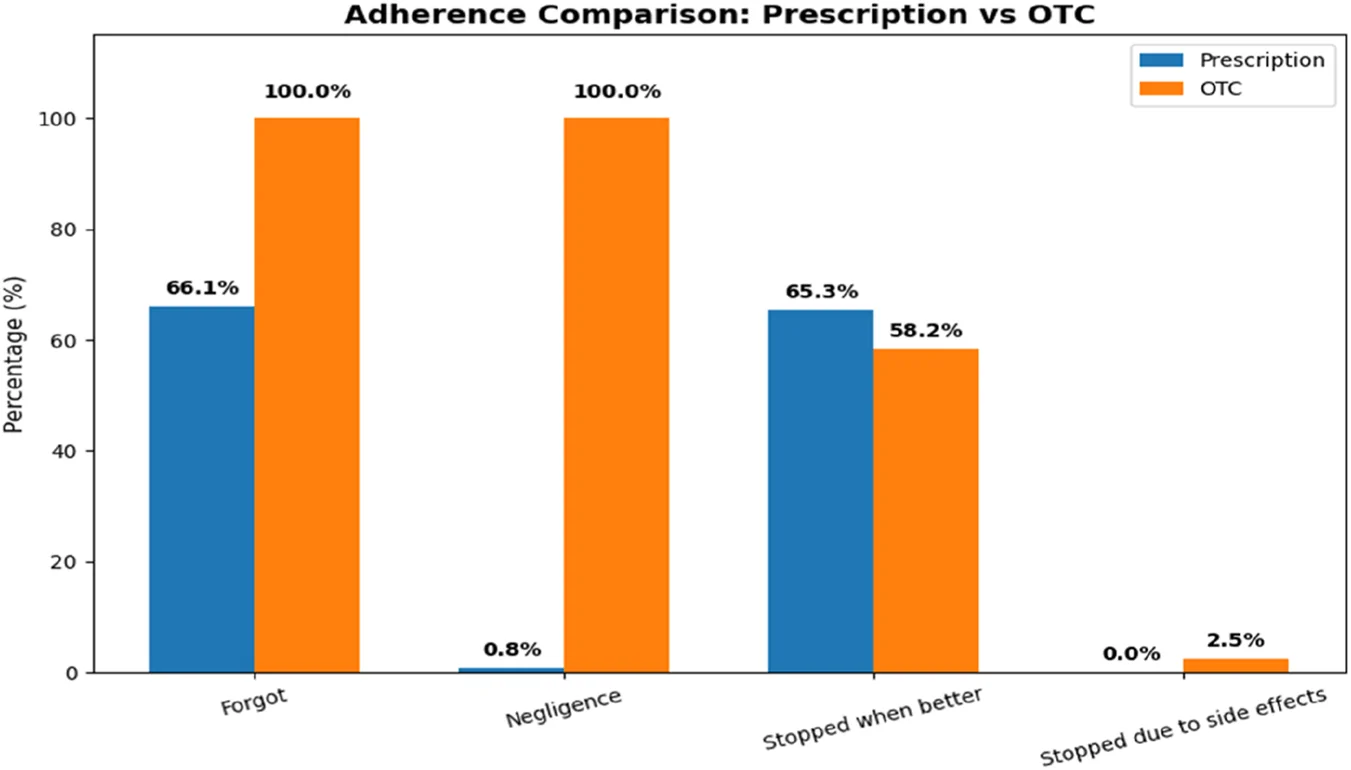
Comparison of medication adherence between prescription and over-the-counter (OTC) mucolytic users. The figure compares self-reported medication adherence between participants receiving prescription and over-the-counter (OTC) mucolytic therapy. Adherence was assessed based on forgetting medication, negligence in taking medication, discontinuation after symptom improvement, and discontinuation due to adverse drug reactions. Overall, participants receiving prescription-guided therapy demonstrated better medication adherence than OTC users across the evaluated domains.

The study findings regarding adherence to treatment indicate that most patients were compliant with their prescribed medications and treatment regimen. Proper adherence was observed to contribute to better symptom control and disease management in respiratory conditions such as Chronic Obstructive Pulmonary Disease, Chronic Bronchitis, and Asthma. However, some patients showed reduced adherence due to factors such as adverse drug reactions, complexity of therapy, and forgetfulness. Overall, adherence to treatment plays an important role in improving therapeutic outcomes and reducing disease complications (Figure 5).

## 5 Discussion

The present study evaluated the pattern and impact of prescription and non-prescription mucolytic agents in the management of respiratory diseases among a community-based population. Respiratory disorders such as cough, bronchitis, asthma, chronic obstructive pulmonary disease (COPD), and upper respiratory tract infections are commonly associated with excessive mucus production, which contributes to airway obstruction and breathing difficulty. Mucolytic agents play an important role in reducing mucus viscosity and improving mucus clearance, thereby enhancing respiratory function and symptomatic relief.

The findings of the study demonstrated that both prescription and non-prescription mucolytics were widely utilized among the study population. Prescription-guided mucolytic therapy was associated with greater symptom improvement, higher medication adherence, and better awareness regarding appropriate medication use than OTC use. These findings are consistent with recent COPD management recommendations that emphasize individualized treatment, adherence support, and regular clinical follow-up (Agustí et al., 2023; Global Initiative for Chronic Obstructive Lung Disease GOLD, 2025; Miravitlles et al., 2022; Buhl et al., 2024; Singh et al., 2025; Cravo et al., 2022). However, these associations should be interpreted cautiously because differences in baseline demographic characteristics, disease severity, healthcare-seeking behaviour, and comorbidity burden may have contributed to the observed outcomes. Prescription mucolytics were predominantly used in patients with chronic or severe respiratory conditions, while non-prescription mucolytics were more frequently used for self-management of mild respiratory symptoms such as productive cough and common cold. This indicates increased public awareness and accessibility of OTC respiratory medications within the community.

Among the prescription mucolytics, agents such as ambroxol, bromhexine, acetylcysteine, and carbocisteine were commonly prescribed by healthcare professionals (Kardos et al., 2021). Their use was mainly associated with conditions requiring long-term respiratory management, including chronic bronchitis and COPD. Prescription use showed better adherence to dosage regimens and physician guidance, which may contribute to improved therapeutic outcomes and reduced complications (Poole and Black, 2001; Stey et al., 2000; Davoodi et al., 2020; Restrepo et al., 2008; Hurst et al., 2010).

In contrast, non-prescription mucolytics, including guaifenesin-containing preparations and cough syrups, were commonly purchased directly from pharmacies without medical consultation. Although these medications provided temporary symptomatic relief, inappropriate self-medication practices were observed in certain individuals, including irregular dosing, prolonged use, and lack of awareness regarding contraindications or potential adverse effects. Such practices may increase the risk of drug misuse and delayed diagnosis of underlying respiratory conditions. Similar studies have highlighted the growing need for pharmacist counselling and regulation of OTC medication use to minimize irrational self-medication practices (World Health Organization, 1998; George et al., 2006).

The study also highlighted that patient preference for OTC mucolytics was influenced by factors such as easy availability, lower cost, convenience, and previous experience with similar medications. However, physician-prescribed mucolytics demonstrated comparatively better clinical improvement due to individualized treatment selection and monitoring. This emphasizes the importance of professional guidance in respiratory disease management.

Adverse drug reactions associated with mucolytic therapy were generally mild and included gastrointestinal discomfort, nausea, throat irritation, and dizziness. Adverse drug reactions observed during the study were predominantly gastrointestinal and dermatological in nature. Common ADRs included nausea, vomiting, abdominal pain, diarrhoea, gastric irritation, rash, itching, dizziness, and throat irritation. Gastrointestinal symptoms such as nausea and vomiting were the most frequently reported reactions. Most ADRs were mild to moderate and manageable, suggesting that the prescribed medications were generally safe and well tolerated. Skin-related reactions such as rash and itching were also observed in a smaller number of patients. Safety monitoring remains important, especially during unsupervised mucolytic use, as inappropriate administration may increase the risk of adverse reactions in vulnerable populations (Chalumeau et al., 2002). Standardized pharmacovigilance terminology such as MedDRA facilitates consistent reporting and comparison of adverse drug reactions across studies (European Medicines Agency, 2024; MedDRA Maintenance and Support Services Organization MSSO, 2024).

Medication adherence was evaluated using a structured self-reported questionnaire specifically designed for this study. Although the instrument allowed practical assessment of medication-taking behaviour in a community setting, it has not undergone formal psychometric validation. Therefore, the adherence findings should be interpreted cautiously and may be influenced by reporting bias. The findings regarding treatment adherence indicated that the majority of patients were compliant with their prescribed therapy. Good adherence is essential in chronic respiratory diseases because regular medication use helps control symptoms, prevent disease progression, and improve quality of life. However, some patients demonstrated reduced adherence due to factors such as adverse drug reactions, forgetfulness, and the complexity of long-term treatment regimens. This highlights the importance of patient counselling, medication education, and regular follow-up to improve adherence and therapeutic outcomes.

The demographic analysis revealed that respiratory diseases and mucolytic use were more prevalent among adults and elderly individuals, particularly smokers and patients exposed to environmental pollutants or occupational dust. This observation supports existing evidence regarding the role of environmental and lifestyle factors in respiratory morbidity (Rogers, 2007; Global Initiative for Chronic Obstructive Lung Disease GOLD, 2023).

Overall, the study suggests that mucolytic agents significantly contribute to symptomatic management and improved quality of life in respiratory diseases (Cazzola et al., 2010; Matera et al., 2020). However, irrational use of non-prescription mucolytics and self-medication practices remain a concern. Community education programs, pharmacist counselling, and stricter regulation of OTC respiratory medications may help promote safe and rational drug use.

Both prescription and non-prescription mucolytics contribute meaningfully to respiratory disease management in community settings. Prescription agents such as N-acetylcysteine and carbocisteine provide greater benefits in chronic and moderate-to-severe disease, particularly in reducing exacerbations. Non-prescription agents like guaifenesin offer accessible symptomatic relief for mild or acute respiratory conditions (Seagrave et al., 2012; Dicpinigaitis and Gayle, 2003). Rational, severity-based selection of mucolytic therapy can optimize patient outcomes and resource utilization in community respiratory care.

These findings align with current international recommendations promoting rational prescribing, patient education, and pharmacist involvement in optimizing respiratory disease management (Agustí et al., 2023; Global Initiative for Chronic Obstructive Lung Disease (GOLD), 2025; World Health Organization, 2022; International Pharmaceutical Federation, 2023). Strengthening patient education and encouraging timely medical consultation may further improve respiratory disease management and optimize therapeutic outcomes in community settings.

## 6 Conclusion

This community-based comparative study evaluated the impact of prescription and non-prescription mucolytic agents on respiratory disease management. The findings demonstrated that prescription-based mucolytic therapy was associated with better symptom improvement, higher treatment adherence, and greater patient awareness regarding appropriate medication use compared with OTC mucolytic use. Patients receiving prescribed therapy benefited from medical supervision, appropriate drug selection, and guidance regarding dosage and duration of treatment. In contrast, self-medication with OTC mucolytics was frequently associated with inadequate knowledge, inconsistent use, and comparatively poorer clinical outcomes. Although adverse drug reactions were generally mild and manageable in both groups, rational use under professional supervision appeared to enhance treatment effectiveness and safety. The study highlights the important role of healthcare professionals and pharmacists in promoting appropriate mucolytic use.

## 7 Limitation of the study

Despite its strengths, this study has some limitations. First, the observational non-randomized study design precludes causal inference and is susceptible to residual confounding despite statistical adjustment. Second, participants were recruited using convenience sampling from selected communities within Pune district. Third, several study variables including medication use, symptom improvement, medication adherence, healthcare utilization, and adverse drug reactions were based primarily on participant self-report and were therefore susceptible to recall bias, reporting bias, and outcome misclassification. Medication adherence to concomitant respiratory therapies was not formally assessed using validated adherence tools. Furthermore, disease severity was not objectively assessed using spirometry, GOLD staging, or validated severity indices, and diagnoses were not confirmed by medical record review for every participant.

## Data Availability

The raw data supporting the conclusions of this article will be made available by the authors, without undue reservation.
